# Clinical characteristics and therapeutic procedure for a critical case of novel coronavirus pneumonia treated with glucocorticoids and non-invasive ventilator treatment

**DOI:** 10.1590/0037-8682-0227-2020

**Published:** 2020-06-01

**Authors:** Juanjuan Zhu, Wei Zhou, Mingyu Zhou, Yang Liu, Jing Yang, Haiyang Li, Xueke Zhao

**Affiliations:** 1 Guizhou Medical University, The Affiliated Hospital of Guiyang, China

**Keywords:** NCP, Glucocorticoid, Non-invasive ventilator

## Abstract

The novel coronavirus pneumonia (NCP) outbreak occurred in Wuhan, China at the end of 2019. Here, we report the clinical characteristics and therapeutic procedure for a case of severe NCP. The patient was started on glucocorticoids and non-invasive ventilator treatment. After treatment, the patient’s symptoms improved, and the status was confirmed as NCP negative. Our results may provide clues for the treatment of NCP.

## INTRODUCTION

At the end of December 2019, the novel coronavirus pneumonia (NCP) outbreak occurred in Wuhan, China. The World Health Organization officially named this new virus as the 2019 novel coronavirus, simply referred to as 2019-nCoV^1^. This virus belongs to the β coronavirus family, and its genetic characteristics are significantly different in Severe Acute Respiratory Syndrome related Coronavirus (SARSr-CoV) and Middle East Respiratory Syndrome related Coronavirus (MERSr-CoV), and it has more than 85% homology with the bat SARS-like CoV^2^. Herein, we report the clinical characteristics and diagnosis of a patient with severe NCP who was treated with glucocorticoids and non-invasive ventilation.

## CASE REPORT

A 61-year-old woman from Guizhou was admitted to the Affiliated Hospital of Guizhou Medical University on January 30, 2020 with one-day history of fever and two-day history of cough. Her body temperature was 37.8^o^C. She was in good health without hypertension, diabetes, and coronary heart disease.

**FIGURE 1:**
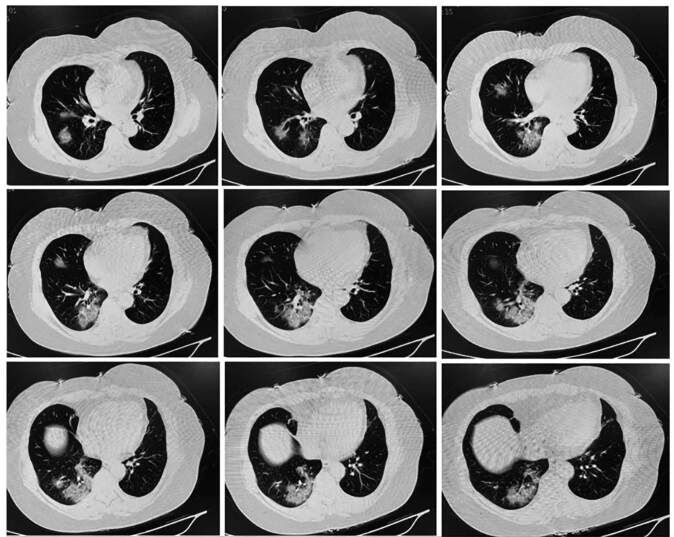
Chest CT image of the patient at admission. Chest CT showed patchy exudates in the lower lobe of right lung, slight fibrosis in the middle lobe of right lung and upper lobe of the left lung, nodules in the upper lobe of the right lung and lower lobe of the left lung and aortic sclerosis.

At admission, her heart rate (85 times/min), respiratory rate (19 times/min) and blood pressure (121/60 mmHg) were in the normal range. Her C-reactive protein (17.38 mg/L) and IL-6 (6.85 pg/mL) levels were elevated. Laboratory examinations showed normal white blood cell count, liver function, kidney function, and procalcitonin level. CT lung imaging showed patchy exudate in the lower lobe of the right lung, slight fibrosis in the middle lobe of the right lung and upper lobe of the left lung, nodules in the upper lobe of the right lung and lower lobe of the left lung, and aortic sclerosis (Figure 1). Additionally, the sputum swab test was performed, and the nucleic acid test indicated positivity for 2019-nCoV.

**FIGURE 2:**
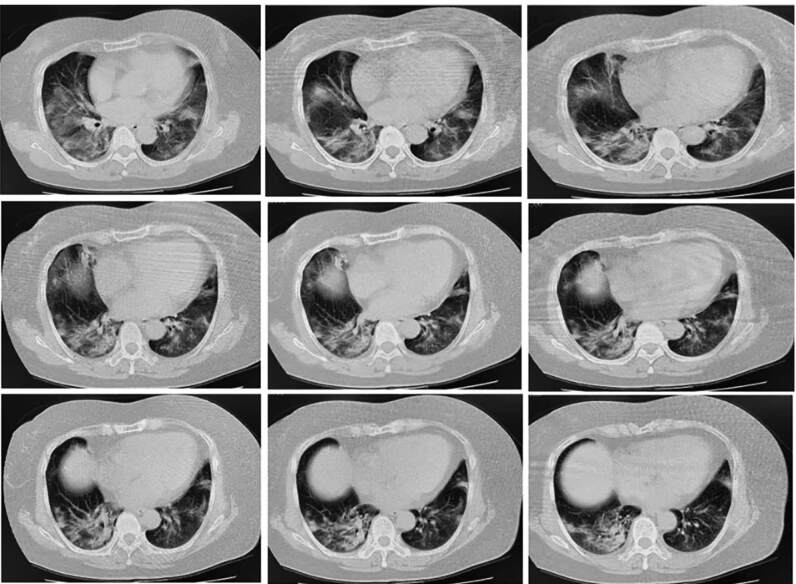
Chest CT image of the patient on February 5, 2020. Chest CT showed obvious exudative lesions in bilateral lungs.

**FIGURE 3:**
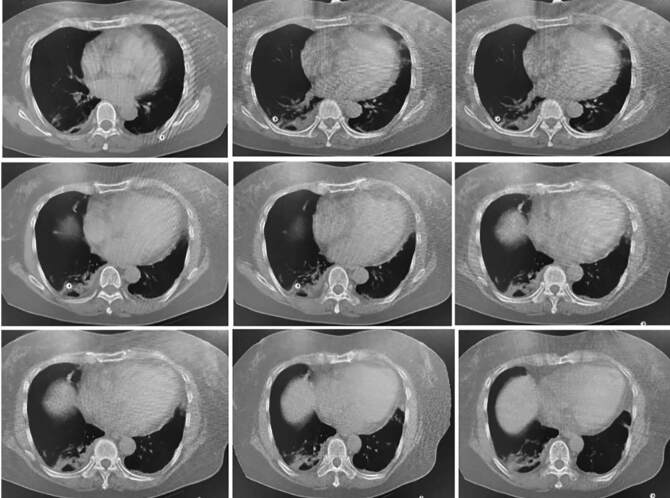
Chest CT image of the patient on February 15, 2020. Chest CT showed absorption of bilateral lung effusion.

Based on her laboratory report, she was treated with lopinavir and ritonavir tablets (two tablets twice a day), arbidol tablets (two tablets three times a day), α-interferon atomization inhalation, *Xuebijing* injection (100 mL twice a day), live combined *Clostridium butyricum* and enterococcus tablets (two tablets three times a day), vitamin C (two tablets three times a day) and 2 L/min oxygen (twice a day, two hours every time) were also administered. On the fourth day of admission, the oxygen saturation of the patient was 89. Considering the severity of the disease, intravenous injection of methylprednisolone (60 mg/d) and subcutaneous injection of thymalfasin (1.6 mg) were administered once a day. On the sixth day of admission, chest CT images revealed obvious exudative lesions in bilateral lungs (Figure 2). On the eighth day of admission, she developed dyspnea and chest distress. Physical exam revealed body temperature of 36.3^o^C, respiratory rate of 20 breaths/min, 3 L/min oxygen by nasal catheter, and oxygen saturation less than 90%. Blood gas analysis showed increased PH (7.58), decreased pO_2_ (58 mmHg), and normal pCO_2_ (43 mmHg). The findings were suggestive of type I respiratory failure with metabolic alkalosis. Subsequently, moxifloxacin and intravenous injection of methylprednisolone (40 mg) were administered, and required assisted respiration with non-invasive ventilator. The dosage of methylprednisolone was reduced every 2-3 days until withdrawal (Table S1). Changes in C-reactive protein and IL-6 levels are shown in Table S2. The results indicated that the levels of IL-6 and C-reactive protein had decreased with the increase in the dosage and duration of methylprednisolone therapy and returned to normal on February 10. In addition, she was placed on non-invasive ventilation four times a day for 2 hours each (Table S3). Blood gas analysis indicated normal pCO_2_ and SpO_2_. However, the value of pO_2_ was lower than normal on February 7 and 8 and returned to normal on February 12 (Table S4). On the 15^th^ day of admission, the patient was administered 2 L/min of oxygen. SpO_2_ was maintained at more than 93%, pO_2_, between 86 and 116 mmHg, and pCO_2_, between 37.5 and 40 mmHg. On the 16^th^ day of admission, chest CT showed absorption of bilateral lung effusion lesions (Figure 3). On the 18^th^ day of admission, she was treated with cefoperazone sodium and sulbactam sodium. On the 19^th^ day of admission, a nasopharyngeal swab was performed for the first time after the treatment and the test result was negative for 2019-nCoV infection. On the 20^th^ day of admission, antiviral drugs and antibiotics were stopped, and she was only treated with thymalfasin and traditional Chinese medicine preparations. Chest CT revealed that the exudation in both lungs was absorbed, and there was a small amount of fibrosis in the middle lobe of the right lung and the upper lobe of the left lung (Figure S1). On the 21^th^ day of admission, a nasopharyngeal swab was performed for the second time after treatment and the test result was negative for 2019-nCoV infection. After consultation with the NCP expert group at our hospital, it was decided that she had reached the relief isolation criteria. Her condition had healed, and she was discharged on 24 February, 2020. It was suggested that she be placed under continued medical observation for 14 days after discharge and return to the hospital for follow-up, two weeks and four weeks after discharge.

## DISCUSSION

Methylprednisolone is one of the glucocorticoids, which is frequently used in clinical settings, it has anti-inflammatory and anti-immune effects and improves airway function^3^. Studies have found that when inflammation occurs in the body, the concentration of glucocorticoids increase in the blood^4^. In the current report, the levels of IL-6 and C-reactive protein decreased with the increase in methylprednisolone dosage, consistent with the above mentioned findings. In addition, the levels of IL-6 and C-reactive protein returned to normal on day 8 of methylprednisolone use. Similarly, Zhou et al. suggested the median time of glucocorticoid use in cured patients was 9.5 days through the observation of critically ill patients with NCP^5^. 

For patients with severe acute respiratory infection and respiratory distress, oxygen inhalation through mask or non-invasive ventilation is necessary^6^. Non-invasive ventilator treatment in the early stage of critical disease can reduce the probability of respiratory failure. In this report, the patient developed type I respiratory failure on day 8 of admission. After nearly one week of intermittent non-invasive ventilator-assisted respiration, the patient’s arterial partial pressure of oxygen, oxygenation index, and oxygen saturation had significantly improved (Table S3 and Table S4). It was found that the use of non-invasive ventilation was effective in improving the patient’s respiratory function.

In summary, we reported the history, diagnosis, and treatment of a patient with NCP. Glucocorticoids and non-invasive ventilator treatment could significantly improve the clinical symptoms in critically ill patients with NCP. It may provide a good reference for the treatment of NCP.
