# Supplementary material for: Clinical characteristics and therapeutic procedure for a critical case of novel coronavirus pneumonia treated with glucocorticoids and non-invasive ventilator treatment
Source: Rev Soc Bras Med Trop. 2020 Jun 1;53:e20200227. doi: 10.1590/0037-8682-0227-2020 (PMC7269524; doi:10.1590/0037-8682-0227-2020)
Supplement: Supplementary file 1 [file 1678-9849-rsbmt-53-e20200227-suppl1.pdf]

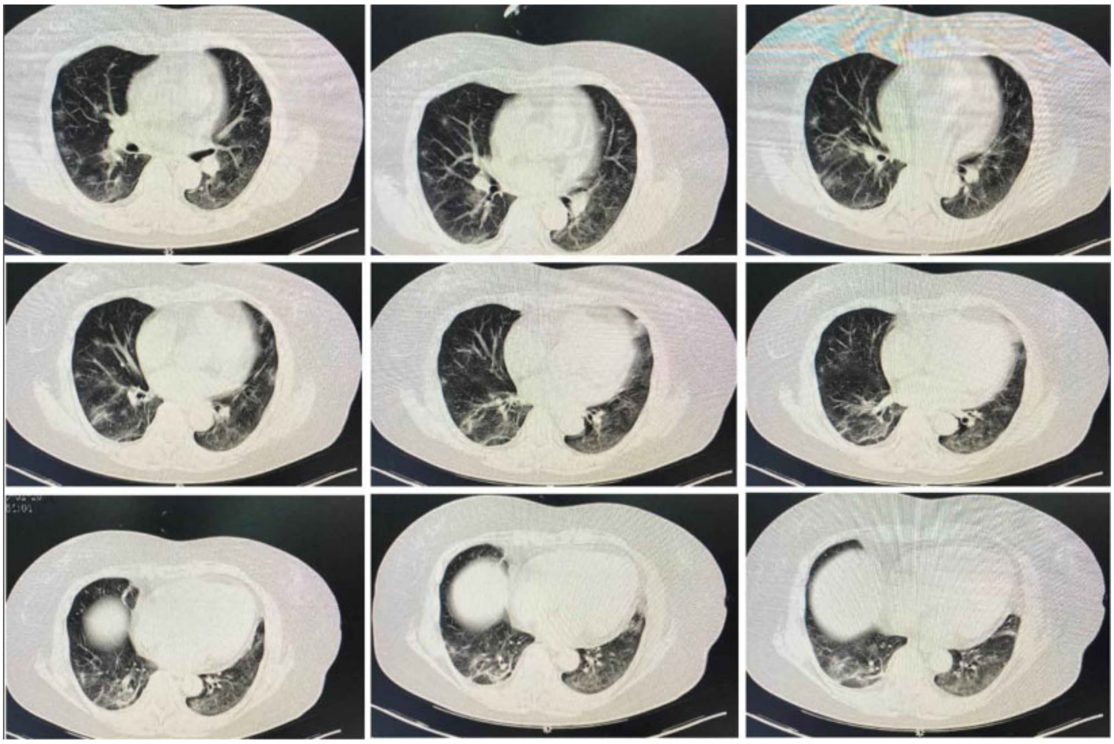

FIGURE S1: Chest CT image of the patient on February 20, 2020

Chest CT revealed absorption of the exudation in bilateral lungs and a small amount of fibrosis in the middle lobe of the right lung and upper lobe of the left lung.

TABLE S1: Time and dose of methylprednisolone

Date	2-7	2-8	2-9	2-10	2-11	2-12	2-13
Dose	40 mg/Q12h	40 mg/Q12h	40 mg/Q12h	20 mg/Q12h	20 mg/Q12h	20 mg/Qd	20 mg/Qd

TABLE S2: The levels of C-reactive protein and IL-6

Date	2-7	2-8	2-9	2-10	2-11	2-12	2-13
C-reactive protein (mg/L)	24.39	35.50	13.03	5.63	2.52	1.32	1.22
IL-6 (pg/mL)	42.11	1.78	2.44	<1.5	<1.5	<1.5	<1.5

TABLE S3: Non-invasive ventilator parameter adjustment (S/T mode)

Date	2-7	2-8	2-9	2-10	2-11	2-12	2-13
IPAP (mmHg)	14	15	15	15	12	12	12
EPAP (mmHg)	8	10	10	10	8	8	8
inspiratory time (sec)	1.2	1.2	1.2	1.2	1.2	1.2	1.2
Rise time (sec)	2	2	2	2	2	2	2
FiO_2	60	60	60	50	40	40	40

TABLE S4: Changes in blood gas analysis

Date	2-7	2-8	2-9	2-10	2-11	2-12	2-13
pO_2 (mmHg)	58.6	79.5	101.5	104	83	102	108.5
pCO_2 (mmHg)	38.6	32	35	34	37.3	38.3	38
SpO_2 (%)	96	94	97	97	96	98	98
